# SLPI deficiency alters airway protease activity and induces cell recruitment in a model of muco-obstructive lung disease

**DOI:** 10.3389/fimmu.2024.1433642

**Published:** 2024-09-05

**Authors:** Ryan Brown, Caoifa Dougan, Peter Ferris, Rebecca Delaney, Claire J. Houston, Aoife Rodgers, Damian G. Downey, Marcus A. Mall, Bronwen Connolly, Donna Small, Sinéad Weldon, Clifford C. Taggart

**Affiliations:** ^1^ Airway Innate Immunity Research (AiiR) Group, Wellcome-Wolfson Institute for Experimental Medicine, School of Medicine, Dentistry and Biomedical Sciences, Queen’s University Belfast, Belfast, United Kingdom; ^2^ Wellcome-Wolfson Institute for Experimental Medicine, School of Medicine, Dentistry and Biomedical Sciences, Queen’s University Belfast, Belfast, United Kingdom; ^3^ Department of Translational Pulmonology, Translational Lung Research Center Heidelberg (TLRC), German Center for Lung Research (DZL), University of Heidelberg, Heidelberg, Germany; ^4^ Department of Pediatric Pulmonology and Immunology, Charité - Universitätsmedizin Berlin, Berlin, Germany; ^5^ Berlin Institute of Health (BIH), Berlin, Germany; ^6^ Patrick G Johnston Centre for Cancer Research, School of Medicine, Dentistry and Biomedical Sciences, Queen’s University Belfast, Belfast, United Kingdom

**Keywords:** respiratory, protease, protease inhibitor, inflammation, chronic disease

## Abstract

Secretory leukocyte protease inhibitor (SLPI) is an important cationic protein involved in innate airway immunity and highly expressed in mucosal secretions, shown to target and inhibit neutrophil elastase (NE), cathepsin G and trypsin activity to limit proteolytic activity. In addition to the potent anti-protease activity, SLPI has been demonstrated to exert a direct anti-inflammatory effect, which is mediated via increased inhibition and competitive binding of NF-κB, regulating immune responses through limiting transcription of pro-inflammatory gene targets. In muco-obstructive lung disorders, such as Chronic Obstructive Pulmonary Disease (COPD) and Cystic Fibrosis (CF), there is an observed elevation in airway SLPI protein concentrations as a result of increased lung inflammation and disease progression. However, studies have identified COPD patients presenting with diminished SLPI concentrations. Furthermore, there is a decrease in SLPI concentrations through cleavage and subsequent inactivation by NE degradation in *Pseudomonas* aeruginosa infected people with CF (pwCF). These observations suggest reduced SLPI protein levels may contribute to the compromising of airway immunity indicating a potential role of decreased SLPI levels in the pathogenesis of muco-obstructive lung disease. The Beta Epithelial Na+ Channel transgenic (ENaC-Tg) mouse model phenotype exhibits characteristics which replicate the pathological features observed in conditions such as COPD and CF, including mucus accumulation, alterations in airway morphology and increased pulmonary inflammation. To evaluate the effect of SLPI in muco-obstructive pulmonary disease, ENaC-Tg mice were crossed with SLPI knock-out (SLPI^-/-^) mice, generating a ENaC-Tg/SLPI^-/-^ colony to further investigate the role of SLPI in chronic lung disease and determine the effect of its ablation on disease pathogenesis.

## 1 Introduction

Secretory leukocyte protease inhibitor (SLPI) is a serine protease inhibitor whose targets include neutrophil elastase (NE), cathepsin G and trypsin. SLPI is expressed at high levels at mucosal surfaces including in the lung and is secreted by neutrophils, macrophages and epithelial cells lining mucus membranes. In addition to its known antiprotease activity, SLPI has also been shown to have both direct anti-inflammatory and anti-microbial activity (1). The anti-inflammatory activity of SLPI is mediated via competitive bidding to the NF-κB consensus binding site, thereby preventing the transcription of pro-inflammatory NF-κB target genes (2). The anti-microbial functions of SLPI are less well understood, however, disruption of bacterial membranes by SLPI’s high positive charge has been postulated as a potential mechanism (3).

In chronic obstructive pulmonary disease (COPD) the concentrations of SLPI in the airways are increased, likely due to the increased protease burden present in these individuals (4). However, it is important to note that in those COPD patients with lower SLPI concentrations, a higher number of exacerbations are observed, suggestive that lower SLPI concentrations potentially increases risk of developing an exacerbation. Importantly, similar correlations between SLPI concentration and exacerbation frequency have been observed in bronchiectasis (5, 6). Additionally, in CF, cleavage and inactivation of SLPI is associated with higher exacerbation rates (7). Degradation of SLPI by NE has also been observed in the lungs of pwCF infected with *Pseudomonas aeruginosa* (8).

Despite the above-mentioned studies linking SLPI with exacerbation rates in several chronic lung diseases, the precise role of SLPI in such diseases remains largely misunderstood. In the present study, we aimed to elucidate the role of SLPI in chronic lung disease. For this, we generated a novel mouse model, where SLPI^-/-^ mice were crossed with βENaC-Tg mice, a model of muco-obstructive lung disease, in which airway specific upregulation of the β subunit of the epithelial sodium channel ENaC leads to airway dehydration, mucus plugging, airway inflammation and lung damage (9, 10). The generation of this mouse model would give us new insights into SLPI function in a chronic disease setting.

## 2 Materials and Methods

### 2.1 Experimental animals

All mice were housed in specific pathogen free (SPF) facilities where housing and experimentation was carried out in accordance with the Animal (Scientific Procedures) Act 1986 and current guidelines approved by the Queen’s University Belfast Ethical Review Committee. The animals were maintained on a 12 hr. cycle of light followed by 12 hr. cycle of darkness with free access to chow and water. ENaC-Tg mice (9) were backcrossed on to the C57BL/6 background as previously described (11). SLPI KO mice, in which the slpi gene was replaced with a neomycin-resistance gene cassette between exons 2-4, were kindly gifted by Koji Atarashi and bred in house (12, 13). SLPI^-/-^ mice were intercrossed with ENaC-Tg mice for 5 generations to generate the double mutant ENaC-Tg/SLPI^-/-^ mice. Mice were sacrificed and samples collected from mice aged up to 14 weeks of age for analysis.

### 2.2 Bronchoalveolar lavage fluid collection and cell counts

Mice were sacrificed by means of an intraperitoneal injection of pentobarbital (Pentoject, Animalcare, York, UK). The lungs were lavaged with 1 ml of sterile saline and the cell-free bronchoalveolar lavage (BAL) fluid was stored at -80°C. Total BAL fluid cell counts were determined using a haemocytometer (Brightline, Harsham, UK) and 0.4% trypan blue (Sigma-Aldrich) to exclude dead cells. Differential cell counts were performed from cytospins (Cytospin 2, Thermo-Shandon, Cheshire, 135 UK) stained with May-Grünwald Giemsa (Merck, Darmstadt, Germany), with a total of at least 400 cells counted per mouse imaged using a Leica DM5500 brightfield microscope (Leica Microsystems, UK).

### 2.3 Zymography

BAL fluid was analyzed under non-reducing conditions on pre-cast Tris-glycine gels containing 0.1% gelatin (Thermo Fisher Scientific). Following electrophoresis, gels were soaked in 2.5% Triton X-100 twice for 30 min each. Gels were then incubated in developing buffer (50 mM Tris-HCl (pH7.5), 200 mM NaCl, 5 mM CaCl_2_, 0.02% Tween) for 30 mins at room temperature before the buffer was refreshed and incubated for 24 hours at 37°C. The gels were stained with Coomassie Blue and destained in 10% acetic acid, 30% methanol under bands were visible. Densitometry was performed on gels using ImageJ software.

### 2.4 Lung histology, airway morphology and mucin gene expression

Histological and morphometric analyses were performed as previously described (9, 10, 14, 15). Lung tissue sections were cut at 6 μm and stained with Alcian blue-periodic acid Schiff (AB-PAS) (Alfa Aesar, Ward Hill, MA) for airway mucus content or haematoxylin and eosin (H&E) (Leica Biosystems Ltd., Newcastle, UK) for destructive index (DI). Airway mucus volume was assessed in the left proximal main axial airways by determining percentage of the airway containing AB-PAS positive material using ImageJ software. DI (a measure of alveolar wall destruction) analysis was performed on five randomly selected frames per mouse at 200x power and analyzed using ImageJ (16). AB/PAS-stained lung section images, airway mucus plugging was quantified by assessing the cross-sectional area of the main axial airway and the luminal area containing mucus using ImageJ 1.54v software (NIH) and Microsoft Excel 2021. DI was determined by means of a 42-point grid. Structures lying under these points were classified as normal or destroyed alveolar and/or duct spaces. Points falling over other structures, such as duct walls, alveolar walls etc. or points falling over positions where the entire structure was not visible were not included in the calculation. Alveolar or duct spaces were classed as normal if surrounded by intact walls or by walls disrupted in only one place. Alveolar spaces were classed as destroyed if the wall of the alveolus was disrupted in 2 or more places or there were 2 or more disruptions of contiguous alveoli that were part of the structures opening onto a single duct system. Duct spaces were classed as destroyed if 2 or more isolated islands of lung parenchyma were observed in the lumen of a duct. Both alveolar and duct spaces were classed as destroyed if the structure was lined by cuboidal epithelium, but was clearly not a normal airway, with or without obvious breaks in the walls or a classic emphysematous lesion was present. DI was calculated as:


(D/D+N)·X·100

D = Destroyed alveolar/duct space

N = Normal alveolar/duct space

A measure of distal airway enlargement, the mean linear intercept (MLI) was calculated by overlaying five horizontal lines onto the H&E images using ImageJ 1.54v software (NIH, Maryland, USA). The count of alveolar walls intersecting the grid formed by these five lines was quantified and averaged for 10 images per mouse, yielding an overall average of intercepts. Subsequently, this average was divided by the length of the grid line using Microsoft Excel 2021 to determine the mean airspace diameter or chord length.

For RNA extraction, lung tissue was homogenized in TRIzol Reagent (Invitrogen) followed by chloroform extraction. Following centrifugation, supernatants were discarded and the pellets were washed in RNA-grade 75% ethanol diluted in DEPC-treated water (Thermo Fisher Scientific). RNA was then quantified using a NanoDrop spectrophotometer (Thermo Scientific) and reverse-transcribed to generate cDNA using a High Capacity Reverse Transcription Kit (Applied Biosystems). Muc5a and Muc5b gene expression analysis was conducted using a TaqMan Gene Expression protocol using Taqman probes (Applied Biosystems) (Muc5ac Mm01276718_m1 and Muc5b Mm00466391_m1). Gene expression was determined by the delta-delta Ct method. Relative fold change of target gene expression was determined by normalization to expression of the reference gene Gapdh (Mm99999915_g1).

### 2.5 Protein extraction and quantification

Lungs were harvested from mice and immediately snap frozen in liquid nitrogen. Frozen lungs were homogenized in 400 μl RIPA buffer supplemented with 100X Halt™ Protease and Phosphatase Inhibitor Cocktail (Thermo Fisher Scientific, Horsham, UK). Homogenates were incubated on ice for 15 min before centrifuging at 17,000 x g for 10 min. Supernatants were collected and stored at -80°C until further use. Protein in lysates were quantified by Bradford assay. Briefly, 2 μl of sample or standard was added to a 96 well plate. A 200 μl aliquot of Bradford reagent (Thermo Fisher Scientific) was added to each well and absorbance was measured immediately at 595 nm (Biotek Synergy HT plate reader). Sample values were measured against the standard curve to determine protein concentration.

### 2.6 Neutrophil elastase activity assay

Neutrophil elastase (NE) activity was determined in mouse BAL fluid using the substrate N-Methoxysuccinyl-Ala-Ala-Pro-Val-7-amino-4-methylcoumarin (AAPV-AMC; Enzo Life Sciences, Exeter, UK) at a final concentration of 20 uM. Experiments were performed ± NE inhibitor N-Methoxysuccinyl-Ala-Ala-Pro-Val-chloromethyl ketone (AAPVCMK) at a final concentration of 1mM. The reaction buffer was 0.1 M Hepes, 0.5 M NaCl, pH 7.5. Samples were incubated in buffer ± inhibitor for 30 min at room temperature before fluorescence (substrate turnover) as determined by excitation at 360 nm and emission at 460 nm was read in a 96-well microplate reader (Synergy HT using Gen5™ software, BioTek UK). Fluorescence emission was read at 1 min intervals for 30 min at 37°C. Results were expressed as the change in relative fluorescence units (ΔRFU) over time.

### 2.7 Western blotting

BAL fluid samples (20μl) were separated on 12% SDS-PAGE gels and transferred onto nitrocellulose membrane (GE Healthcare, Buckinghamshire, UK). Membranes were blocked and incubated with TIMP-1 antibody (Bio-techne (AF980) 1:2000) or SLPI antibody (R&D Systems (AF1735), 1:2000) overnight at 4°C. Membranes were washed, incubated with HRP-conjugated secondary antibodies, developed using chemiluminescent substrate (Western Lightning, PerkinElmer, Coventry, UK) and viewed using Syngene G:Box and GeneSnap software (Syngene, Cambridge, UK). Densitometry was performed on blots using GeneTools software (Syngene).

## 3 Human sample collection and processing

Human sputum samples were obtained as part of the AIRSPACE (Assessing the Inflammatory and microbial ReSPonse to intravenous Antibiotics in Cystic fibrosis pulmonary Exacerbations) study. The study was approved by the London-Riverside Research Ethics Committee (19/LO/0811) (17). Sputum plugs were weighed and re-suspended in a volume of Dulbecco’s Phosphate Buffered Saline (PBS) equal to 8 times the weight of the sputum plugs and then homogenized by repeat pipetting with a plastic transfer pipette and vortexed for 15 s. The mixture was placed on a bench rocker for 15 minutes on ice and then centrifuged at 790 x g for 10 min at 4°C. A volume of supernatant equal to four times the weight of the sputum plugs was removed and centrifuged at 1500 x g for 10 min at 4°C. The cell-free supernatant was stored at -80°C until use.

### 3.1 SLPI and TIMP-1 ELISAs

Sputum supernatant SLPI levels (R&D Systems DY1274-05) and TIMP-1 (R&D Systems DY970) were quantified by in sputum by ELISA as per the manufacturer’s instructions. A 1:10 dilution of processed sputum was used for the SLPI ELISA and undiluted processed sputum for the TIMP-1 ELISA.

### 3.2 Statistics

All data were analyzed using GraphPad Prism 8 (GraphPad Software Inc., San Diego, CA) and are reported as mean ± SEM. Statistical analysis was performed using unpaired two-tailed t-test or one way ANOVA with Tukey’s multiple comparisons test for normally distributed data and two-tailed Mann-Whitney test or Kruskal-Wallis test for non-normally distributed data as appropriate. P < 0.05 was accepted to indicate statistical significance; *p< 0.05, **p< 0.01, ***p< 0.001.

## 4 Results

### 4.1 Upregulation of SLPI in the ENaC-Tg lung

**Figure 1:**
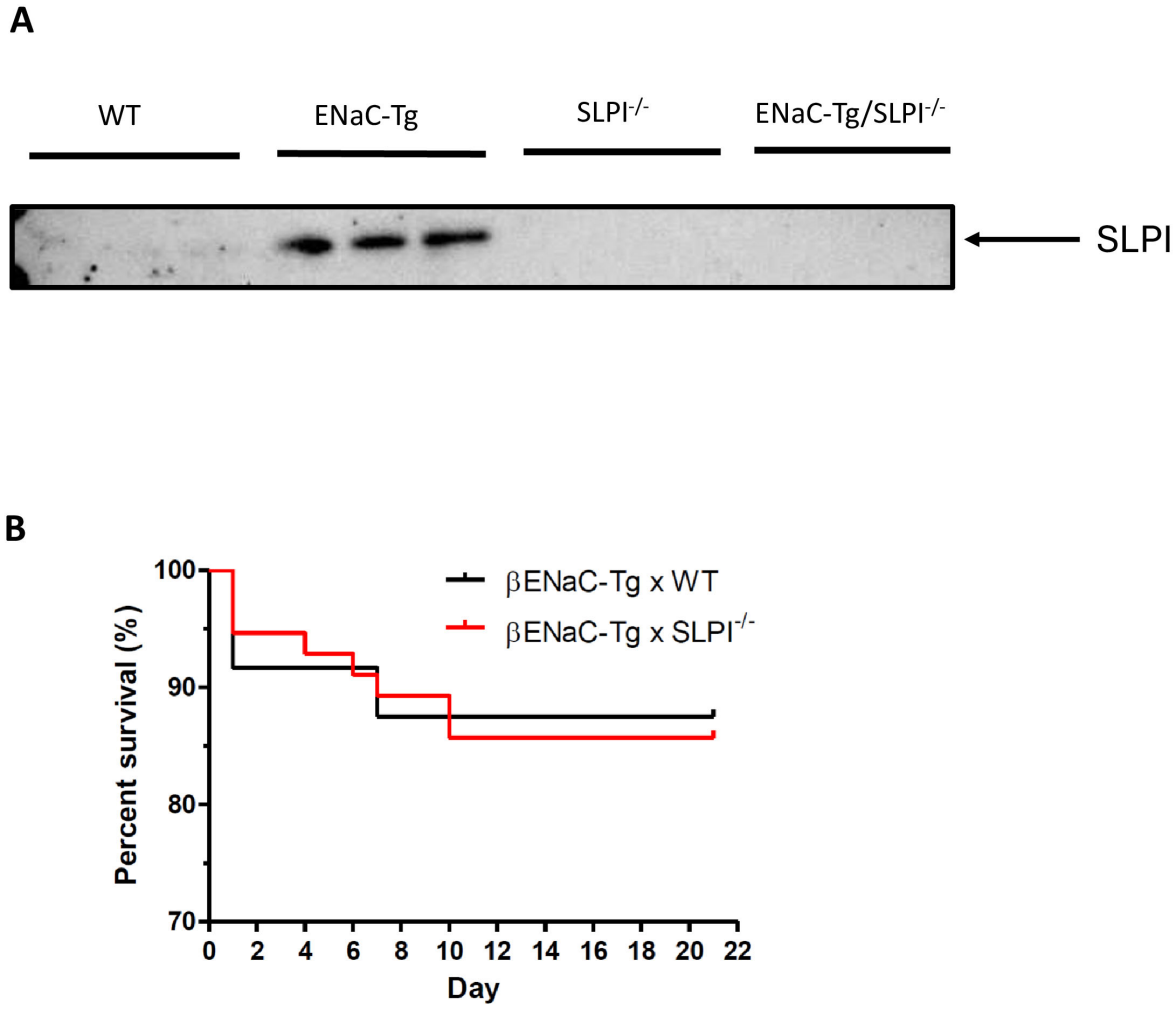
**(A)**. SLPI is increased in ENaC-Tg mice. SLPI was evaluated by Western blot of BAL fluid from WT, ENaC-Tg, SLPI^-/-^ and ENaC-Tg/SLPI^-/-^ mice. **(B)**. Survival curves of ENaC-Tg and ENaC-Tg/SLPI^-/-^ mice demonstrated no differences in survival between both mouse lines.

To determine the validity of ENaC-Tg mice as a model to assess the effects of targeting SLPI, we examined SLPI protein levels in BAL fluid in mice by western blot (**Figure 1A**). We observed increased levels of SLPI in the BAL fluid from ENaC-Tg mice, compared to that of WT mice. SLPI was completely absent in SLPI^-/-^ and ENaC-Tg/SLPI^-/-^ mice, as expected. This demonstrates that increased SLPI is a feature of ENaC-Tg mice, while also confirming the lack of SLPI protein in SLPI^-/-^ mice. ENaC-Tg mice raised on the C57Bl/6 background have a ~20% mortality rate during the first days of life as a result of airway mucus plugging and asphyxiation. Knockout of SLPI had no effect on this early life mortality in ENaC-Tg mice, suggesting that loss of host SLPI does not affect the development of airway mucus plugging during the first days of life in this model, which is thought to cause asphyxiation and early death in this model (**Figure 1B**).

### 4.2 Ablation of SLPI reduces airway mucus plugging in ENaC-Tg mice but not lung damage

**Figure 2:**
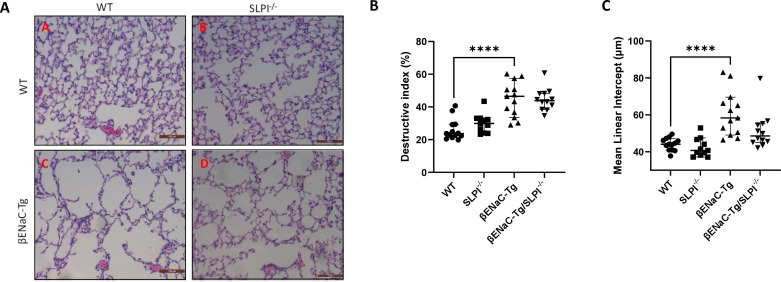
Lung damage is unaffected by SLPI knockout in ENaC-Tg mice. Lung damage was not significantly different between ENaC-Tg and ENaC-Tg/SLPI^-/-^ mice as determined histologically **(A)** and by measurement of destructive index (DI) **(B)** and mean linear intercept **(C)**. ****p<0.0001. Scale bars = 100μM.

**Figure 3:**
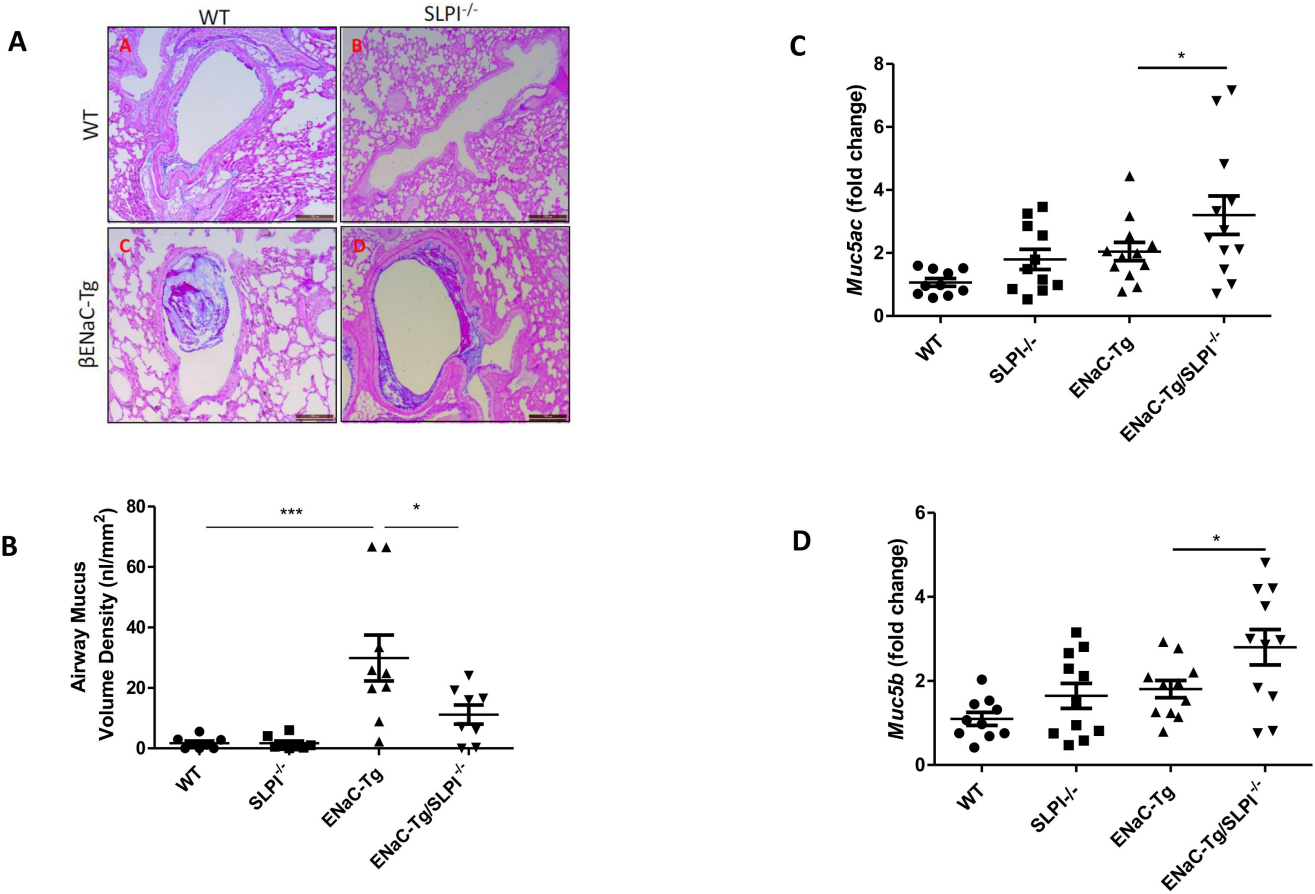
Airway mucus plugging is reduced in ENaC-Tg/SLPI^-/-^ mice. Mucus accumulation in the airway was decreased in the ENaC-Tg/SLPI^-/-^ versus ENaC-Tg mice as determined by AB-PAS staining **(A)** and by measurement of Airway Mucus Volume **(B)**. Muc5a **(C)** and Muc5b **(D)** gene expression was increased in ENaC-Tg/SLPI^-/-^ versus ENaC-Tg mice. *p<0.05, ***p<0.001. Scale bars = 200μM.

SLPI ablation in ENaC-Tg mice did not affect lung damage as shown in **Figures 2A–C**. As expected, there was a significant increase in DI and MLI in the ENaC-Tg mice compared to WT mice, but the absence of SLPI in the ENaC-Tg mouse did not significantly alter the DI or MLI. Airway mucus plugging is a key pathological feature of ENaC-Tg mice occurring from birth. Histological analysis of AB-PAS-stained images revealed that ablation of SLPI resulted in a decrease in airway mucus plugging in ENaC-Tg mice (**Figures 3A, B**). Interestingly, expression data from whole lung showed increased expression of the major mucin *Muc5ac* and *Muc5b* genes in ENaC-Tg/SLPI^-/-^ mice compared to ENaC-Tg mice (**Figures 3C, D**). This increase in mucin gene expression may be part of a mechanism to compensate for mucin protein reduction in the absence of SLPI (**Figure 3B**).

### 4.3 Ablation of SLPI increases pulmonary inflammatory cell recruitment in ENaC-Tg mice

**Figure 4:**
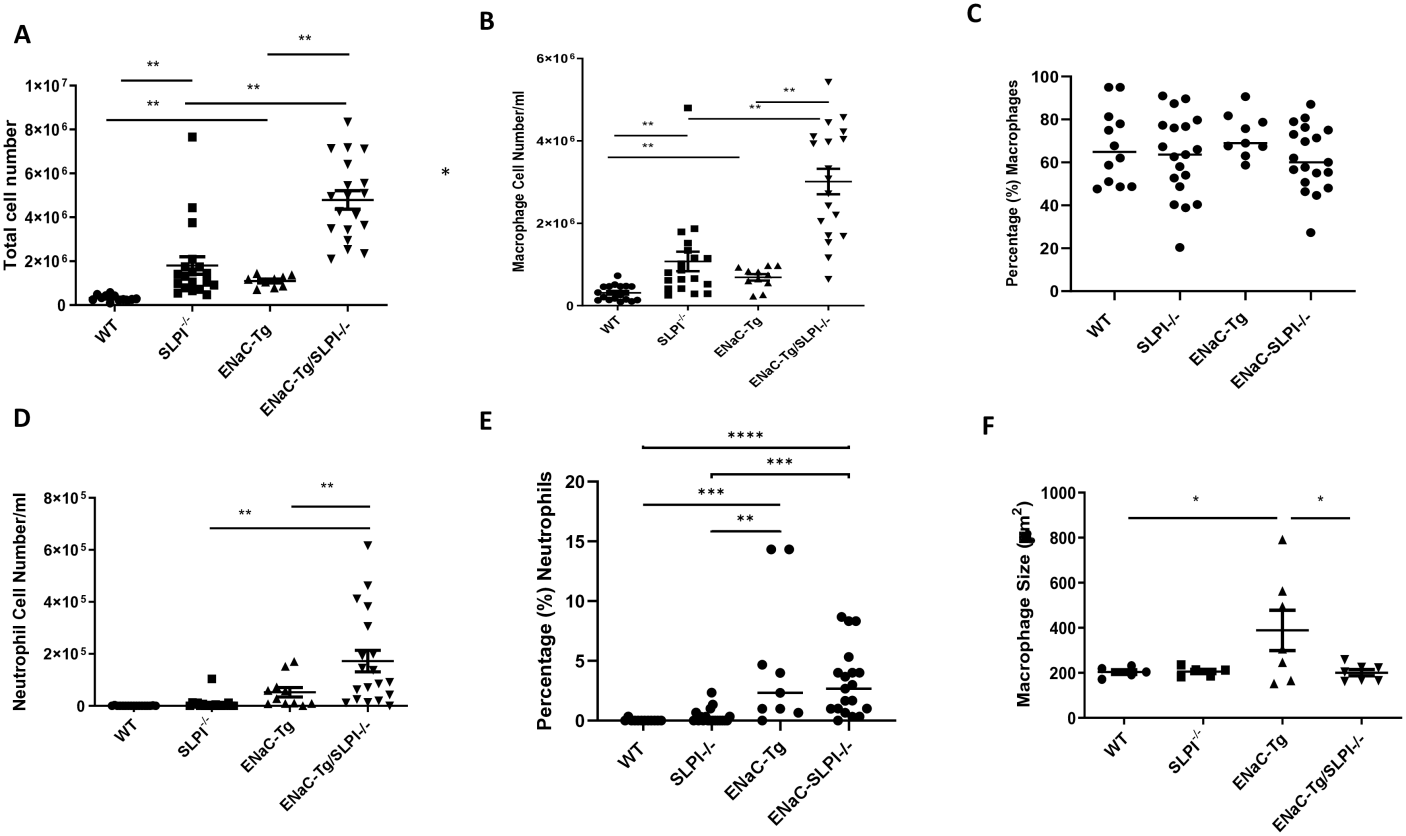
Evaluation of BAL total inflammatory cell counts **(A)**, macrophages **(B)**, percentage macrophages **(C)**, neutrophils **(D)**, percentage neutrophils **(E)** and macrophage size **(F)** in WT, ENaC-Tg, SLPI^-/-^ and ENaC-Tg/SLPI^-/-^ mice. *p<0.05, **p<0.01, ***P<0.005, ****P<0.0001.

Inflammatory cell infiltration into the airways is a key feature of muco-obstructive lung diseases. Indeed, ENaC-Tg mice present with increased total inflammatory cell counts, followed by significant neutrophilia in adult mice. To examine the effects of SLPI loss on pulmonary inflammation, we assessed immune cell numbers in BAL fluid. As expected, adult ENaC-Tg mice presented with significant elevated cell infiltrate (**Figure 4A**). Ablation of SLPI resulted in significantly increased macrophage infiltration in both WT and ENaC-Tg mice (**Figures 4B, C**) with a significant increase in neutrophils in ENaC-Tg/SLPI^-/-^ mice alone (**Figures 4D, E**). Airway macrophage size is increased in ENaC-Tg mice compared to WT mice which we have also demonstrated (**Figure 4F**). However, macrophage size in ENaC-Tg/SLPI^-/-^ mice was significantly reduced and similar to that observed in WT or SLPI^-/-^ mice (**Figure 4F**).

### 4.4 Genetic ablation of SLPI alters lung MMP activity in βENaC-Tg mice

**Figure 5:**
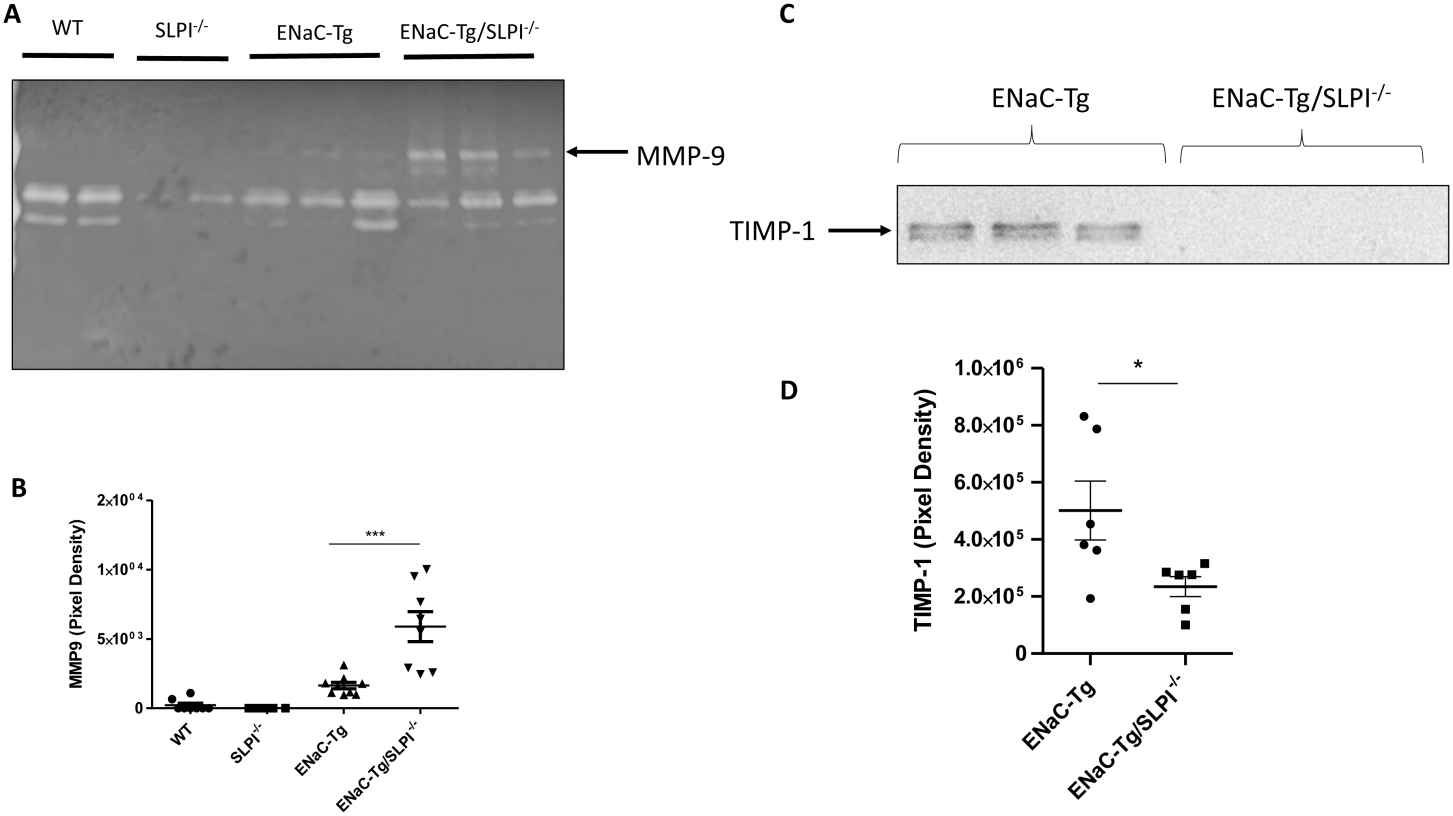
SLPI knockout alters BAL fluid MMP-9 activity **(A, B)** and TIMP-1 **(C, D)** in ENaC-Tg mice. *p<0.05, ***p<0.005.

To determine if ablation of SLPI altered airway protease activity, we examined protease activity in the BAL fluid by gelatin zymography (**Figure 5A**). We observed the presence of banding at ~92 kDa representing MMP9 activity. Densitometry revealed significant increases in MMP9 activity in ENaC-Tg/SLPI^-/-^ mice, compared to that of ENaC-Tg mice (**Figure 5B**). Soluble NE activity was undetectable in the BAL of all four genotypes (data not shown). This was not unexpected, as previously published data has demonstrated that NE activity is present on the surface of neutrophils but is undetectable as free NE in the BAL fluid (18).

### 4.5 Genetic ablation of SLPI alters airway protease/anti-protease balance in βENaC-Tg mice

To examine if changes to lung protease and anti-protease protein concentrations were responsible for the observed changes in MMP-9 activity in ENaC-Tg/SLPI^-/-^ knockout mice, we analyzed endogenous MMP inhibitor TIMP-1 by western blot on lung tissue (**Figure 5C**). There was a significant reduction in protein levels of the MMP inhibitor TIMP-1 in ENaC-Tg/SLPI^-/-^ mice compared to ENaC-Tg mice (**Figures 5C, D**). These data suggest that increases in MMP-9 activity are potentially due to decreased levels of the inhibitor TIMP-1.

### 4.6 SLPI and TIMP-1 correlate with NE in human CF sputum

**Figure 6:**
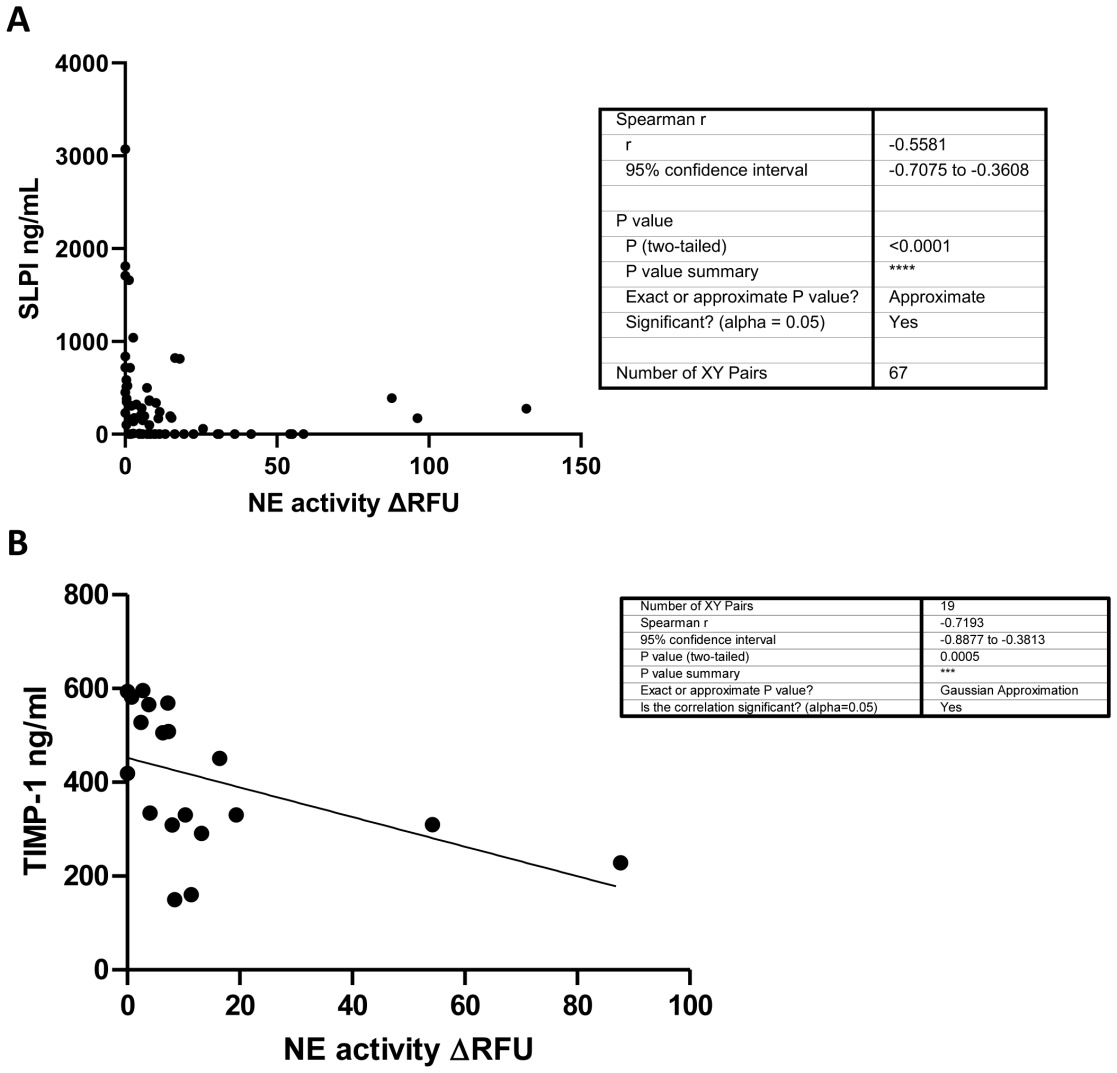
SLPI **(A)** and TIMP-1 **(B)** negatively correlate with NE activity in human CF sputum. NE activity and SLPI and TIMP-1 levels (ELISA) were measured in CF sputum from pwCF. ***p<0.0005, ****P<0.0001.

While we could not detect free NE in BAL samples from ENaC-Tg mice, it is recognized that SLPI is an endogenous inhibitor of NE (19). Therefore, we analyzed sputum from pwCF, and demonstrated that SLPI negatively correlates with NE activity (**Figure 6A**). Furthermore, NE activity in CF sputum negatively correlates with TIMP-1 levels (**Figure 6B**). Indeed, previously published data indicates that NE is capable of cleaving TIMP-1 (20). These data highlight a potential mechanism by which SLPI deficiency in ENaC-Tg mice can lead to reduced TIMP-1 protein levels and increased MMP-9 activity as a result of increased cleavage of TIMP-1 by NE.

## 5 Discussion

SLPI is a potent protease inhibitor highly expressed in airway mucosal secretions, exerting additional direct and indirect anti-inflammatory properties as part of the innate immune response in the airways. However, decreasing airway concentrations of SLPI are associated with the progression of various lung pathologies (5, 21). In this study, the role of SLPI in the pathogenesis of chronic lung disease was evaluated in ENaC-Tg mice – a model of muco-obstructive pulmonary disorders.

In this study, we show that genetic ablation of SLPI alters airway protease activity, mucus plugging and pulmonary inflammation, without affecting the development of lung damage. We speculate that changes to lung inflammation and mucus clearance may be due to increased protease MMP activity in the lungs of ENaC-Tg/SLPI^-/-^ mice and decreased TIMP-1. Unrestrained protease activity has been linked to the pathogenesis of chronic lung disease in a number of previous studies in the ENaC-Tg model (14, 18, 22). However, whether knockout of a single protease inhibitor could exacerbate ENaC-Tg pathogenesis has not previously been studied.

These observed alterations in altered airway protease/antiprotease balance (increased MMP-9 and decreased TIMP-1) may be attributed, at least in part, to increased NE activity in the absence of SLPI. Elevated NE is linked to decreased levels of TIMP-1 through degradation, a process to which MMP-9 appears resistant (20, 23). In addition, increased NE activity has been demonstrated to facilitate and enhance activation of pro-MMP-9, further promoting MMP-9-associated protease activity in the airways (24). In the ENaC-Tg phenotype, previous literature has demonstrated that NE activity is predominantly localized to neutrophils as surface-bound-NE, while free NE is considered undetectable in the BAL fluid of the ENaC-Tg model (18). In addition, neutrophil-bound NE activity has been shown to be increased in lung neutrophils isolated from the airways of pwCF (25). Therefore, to determine the inhibitory potential of SLPI on NE and the subsequent relationship with airway protein degradation, concentrations of SLPI and TIMP-1 were quantified and correlated against NE elastase activity in CF sputum. Airway protein levels of both inhibitors were observed to negatively correlate with NE activity in CF patients, suggesting that as SLPI concentrations decrease, NE protease activity increases, which subsequently may promote TIMP-1 degradation in these samples, in agreement with previous studies (20). These results further elucidate the potential mechanism by which SLPI regulates protease activity in innate airway immunity and muco-obstructive lung disease. Therefore, unchecked NE activity on the surface of neutrophils in airways of the ENaC-Tg/SLPI^-/-^ mice (due to the absence of SLPI) may increase degradation of TIMP-1 while also promoting the activation of MMP-9. We did not observe changes in lung damage despite changes in airway protease activity. A wide range of proteases and inflammatory mediators have been shown to contribute to airway damage in ENaC-Tg mice including Cathepsin S, NE, MMP-12 and eosinophil products (14, 18, 22, 26). Therefore, it is possible that a deficiency of one protease inhibitor, such as SLPI, is not sufficient to alter the development of lung damage.

Recent findings have demonstrated that NE can also be bound to exosomes secreted by neutrophils and in this format is resistant to inhibition by endogenous inhibitors such as alpha 1-antitrypsin (27–29). It isn’t clear if smaller endogenous NE inhibitors such as SLPI can inhibit this exosome-bound version of NE but this is clearly worth evaluating in future studies. Another potential downstream consequence of NE activity is the generation of collagen-derived matrikine, Pro-Gly-Pro (PGP) which is a known chemoattractant and may contribute, in part, to the increased cell recruitment observed in our SLPI deficient mouse model (30, 31).

We also observed changes in the recruitment of macrophage-like cells in both WT and ENaC-Tg mice. This finding suggests that these changes are independent of the muco-obstructive phenotype as mucus was decreased in the ENaC-Tg/SLPI-/- mice. Interestingly, macrophages in BAL from ENaC-Tg/SLPI^-/-^ are significantly smaller than those from ENaC-Tg mice. Monocytic cells in ENaC-Tg mice are characterized by enlarged and highly vacuolated cells (32). Macrophage size has previously been shown to increase following activation, therefore, the smaller cell size may suggest a reduction in the activation of these cells in ENaC-Tg/SLPI^-/-^ mice (33). The smaller size of macrophage-like cells in SLPI-/-mice may point towards increases in recruited monocytes rather than activated alveolar or tissue-resident macrophages. Further work will be needed to confirm the activation state and source of the macrophages in the ENaC-Tg/SLPI^-/-^ airways.

Finally, the finding that SLPI, probably indirectly, regulates the MMP/TIMP ratio indicates that SLPI’s role extends beyond the direct inhibitory impact it has on neutrophil serine proteases such as NE and highlights its importance as a major protease regulator as it has previously been shown to also regulate the expression and activity of cysteinyl cathepsins (34). Taken together, this study shows that SLPI contributes to the regulation of protease activity and airway monocyte recruitment in both WT and ENaC-Tg mice which may also impact on mucus plugging.

## Data Availability

The raw data supporting the conclusions of this article will be made available by the authors, without undue reservation.
